# Factors associated with poor prognosis in elderly biopsy-only glioblastoma patients

**DOI:** 10.1007/s00701-025-06681-7

**Published:** 2025-10-11

**Authors:** Jana Edriss, Julie Cheung, Erik Kronvall, Henrietta Nittby Redebrandt, Erik Uvelius

**Affiliations:** 1 Department of Neurosurgery, Institution of Clinical Sciences, Lund University, Lund, Sweden; 2 Department of Neurosurgery, Karolinska University Hospital, Stockholm, Sweden; 3 Department of Clinical Neuroscience, Karolinska Institute, Stockholm, Sweden

**Keywords:** Glioblastoma, Brain tumor, Biopsy, Oncology treatment, Palliative care, Elderly

## Abstract

**Purpose:**

Glioblastoma (GBM) in elderly patients has a poor prognosis. About one-third of patients have impaired perioperative performance status (PS) and often excluded from clinical trials. Brain biopsy is the standard diagnostic approach when resection is not feasible. Previous studies on preoperative prognostic factors have mostly focused on resected patients. This study aimed to identify preoperative factors associated with reduced three-month survival and treatment incompletion in elderly biopsy-only patients, with the three-month endpoint reflecting early mortality and rapid disease progression that often prevents treatment completion.

**Methods:**

We retrospectively reviewed biopsy-only GBM patients aged > 65 years between 2017 and 2020. Preoperative prognostic factors were analyzed using logistic regression, and overall survival (OS) was estimated using Kaplan–Meier.

**Results:**

A total of 132 patients were included. Median OS was 4.6 months, and 50% completed treatment. Palliative treatment was given to 17% of patients (median OS 1.3 months). Poor PS (OR = 0.2), larger tumor volume (OR = 0.9), and central tumor location (OR = 0.3) were independently associated with reduced three-month survival. Poor PS was the only predictor of treatment incompletion (OR = 0.06); in this subgroup, The median OS was 1.6 months, with only one of 21 completing treatment.

**Conclusion:**

In elderly patients with biopsy-only GBM, poor preoperative PS, central tumor location, and larger tumor volume were significantly associated with reduced short-term survival. Patients with poor preoperative PS were also less likely to complete treatment. These findings may aid in counseling on the potential benefits of biopsy in this vulnerable group.

## Introduction

Glioblastoma (GBM) is the most common and aggressive primary brain tumor, with a median overall survival (OS) of 15 months and a 5-year survival rate of only 5% [6, 34, 47]. Incidence increases with age, peaking between 75 and 84 years [39]. Elderly patients face a worse prognosis, with a 5-year survival rate of only 2.1% despite receiving multimodal treatment [6, 35].

Standard treatment prioritizes maximal safe tumor resection as increased extent of resection is associated with improved progression-free survival (PFS) and OS [12, 16, 17]. However, in some cases, resection is considered unfeasible or unsafe due to factors such as tumor involvement in eloquent brain regions or patient-related comorbidities, in which only a biopsy is performed to confirm the diagnosis [2]. While generally safe, biopsy carries a mortality risk of 0.6–6.8% and morbidity of 3–13% [50]. Vulnerable patient groups, including those aged over 65 years and those with infratentorial or centrally located tumors, face higher complication rates [28, 30, 33]. The OS for biopsy-only patients, who represent 15–30% of all GBM cases, is significantly lower than for those undergoing resection [10].

Following biopsy or resection, oncological treatment typically involves radiotherapy (RT) combined with concurrent and adjuvant temozolomide (TMZ) chemotherapy, as defined by the Stupp regimen [40]. Chemotherapy are guided by O6-methylguanine-DNA-methyltransferase (MGMT) promoter methylation status, patient age and functional status, commonly assessed using the Eastern Cooperative Oncology Group (ECOG)/World Health Organization performance status (WHO PS) scale [8, 10]. While combination Therapy continues to offer a statistically significant survival benefit in patients over 70 years, the effect is less pronounced in this age group [18].

Despite the high proportion of elderly GBM patients undergoing biopsy-only procedures, their short-term survival benefit remains unclear. This retrospective, single-center cohort study aimed to assess survival outcomes and sought to identify preoperative factors associated with reduced three-month survival and the inability to complete oncological treatment.

## Methods and materials

### Study cohort

Patients aged 65 years or older who underwent brain biopsy for suspected GBM were retrospectively identified through surgical logs, from 2017 to 2020 at the Department of Neurosurgery, Skåne University Hospital, Lund, Sweden. Inclusion criteria were: (I) patient age over 65 years, (II) preoperative neuroradiological suspicion of primary GBM, (III) biopsy-only procedure, and (IV) histopathological confirmed IDH-wildtype (wt) GBM [41]. Patients with a previously verified brain tumor and those who underwent tumor resection following biopsy were excluded.

Ethical approval was obtained by the Swedish Ethical Review Authority board (Dnr 2023–02584-01 (Nittby Redebrandt). Informed consent was waived in accordance with the ethical review to reduce bias by only including those alive and able to provide informed consent retrospectively.

### Clinical variables

Patient data were collected from electronic medical records at Skåne University Hospital on the day of neurosurgical admission. Patient-related variables included age, sex, Charlson Comorbidity Index (CCI) [22], and clinical status according to the WHO PS [5]. Neurological status prior to surgery was assessed using the Neurologic Assessment in Neuro-Oncology (NANO) scale [27]. This scale is based on direct physician observations, evaluating patients in nine domains: gait, strength, upper extremity ataxia, sensation, visual fields, facial strength, language, level of consciousness, and behavior. Each domain is scored from 0 to 3 or 0 to 2, depending on the specific category, with higher scores indicating greater neurological impairment. Furthermore, radiographic tumor characteristics such as tumor multifocality, midline shift, tumor volume, affected lobes, and central tumor growth were noted. All patient scans were reviewed by a consultant neuroradiologist prior to surgery. Cerebral edema and corticosteroid effects were assessed based on patient symptoms. Multifocal tumors were defined as ≥ 2 separate contrast-enhancing lesions. Tumor volume was calculated using the ellipsoid geometric formula (v = abc/2). Centrally located tumors were defined as lesions involving deep brain structures, including the basal ganglia, thalamus, brainstem, and periventricular zones [24]. Tumors with partial involvement of these areas were also classified as central if the predominant tumor mass was located within these regions, based on radiological assessment.

All patients received an integrated histopathological diagnosis by a consultant neuropathologist. While most diagnoses were made according to The 2016 WHO classification, conversion to The 2021 criteria [41] did not affect inclusion, as all tumors were IDH-wt. Treatment decisions were made by a multidisciplinary team (MDT) following biopsy, which evaluated each case based on tumor and patient characteristics in accordance with current clinical guidelines [26]. Data on treatment modalities, including palliative care, RT, TMZ, and combination therapy, as well as treatment completion status, were recorded. Treatment completion was defined as receiving six cycles of TMZ after RT in the combination group, and six cycles of TMZ alone in the monotherapy group.

### Statistical methods

All statistical analyses were performed using R (version 4.2.1) [31], utilizing the following packages ‘ggfortify’ [15], ‘survival’ [42] and ‘tidyverse’ [49]. Descriptive data for categorical variables were presented as numbers and proportions (n, %). The normality of continuous variables was assessed using the Shapiro–Wilk test, with a p-value < 0.05 indicating non-normality. Continuous variables were reported as medians and interquartile ranges (IQR). Survival analysis was performed using the Kaplan–Meier (KM) method to estimate median OS. Differences between groups were assessed using the log-rank test. Logistic regression models were used to identify preoperative factors associated with three-month postoperative survival and completion of oncological treatment. Factors with p-value < 0.1 in univariable analyses were included in multivariable analyses. Age and sex were included as confounders regardless of significance in univariable analyses. Missing data were handled by listwise deletion for each specific analysis. P-values < 0.05 were considered statistically significant.

## Results

### Patient characteristics and survival

**Fig. 1:**
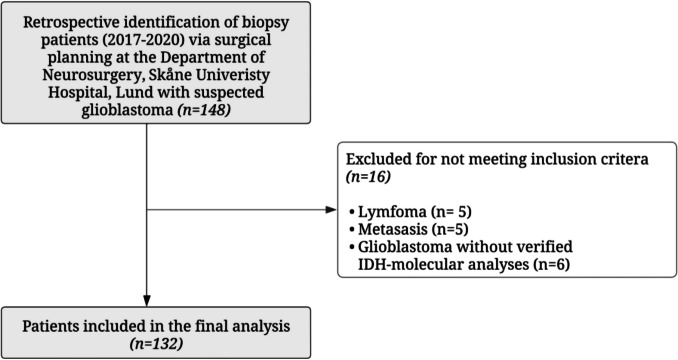
Flow chart of the patient selection

The initial cohort consisted of 148 patients who underwent brain biopsy for magnetic resonance imaging (MRI) findings indicative of GBM. Histopathological assessment revealed alternative diagnoses in 10 patients: five had lymphoma and five had brain metastases. In six additional patients, histopathology was consistent with GBM but lacked IDH classification. As our study focused on IDH-wt GBM, These 16 patients were excluded, resulting in a final cohort of 132 patients (Fig. 1).

Baseline characteristics of the study population are summarized in Table 1. The median age was 74 years (IQR: 70–77), and 75 patients (57%) were male. A total of 111 patients (84%) presented with a WHO PS score of 0–2, while 21 patients (16%) had a PS of 3–4. Centrally located tumors were observed in 98 patients (74%), and 57 patients (43%) had multifocal tumors.

**Table 1:** Descriptive baseline characteristics of patient cohort (*n* = 132)

Preoperative predictors	Baseline parameters	*n* (%)	Completed	Survival at 3 months
		132 (100)	65 (59.2)	83 (62.8)
Sex	Male	75 (56.8)	32 (24.2)	46 (34.8)
	Female	57 (43.2)	33 (25.0)	37 (28.0)
Age				
	Median [IQR]	74 [70–77.3]		
	≤ 75	72 (54.5)	42 (31.8)	51 (38.6)
	> 76	60 (45.5)	23 (17.4)	32 (24.2)
CCI				
	Mild (0–3)	60 (45.5)	29 (22)	40 (30.3)
	Moderate (4–5)	45 (34.1)	22 (16.7)	26 (19.7)
	Severe (6–7)	20 (15.2)	11 (8.3)	12 (9.1)
	Very severe (> 8)	9 (6.8)	3 (2.3)	5 (3.7)
WHO PS				
	0–2	111 (84.1)	64 (48.5)	79 (59.8)
	3–4	21 (15.9)	1 (0.8)	4 (3.0)
NANO				
	≤ 2	49 (37.1)	33 (25.0)	40 (30.3)
	3	18 (13.6)	7 (5.3)	8 (6.1)
	4–5	33 (25.0)	16 (12.1)	21 (15.9)
	6 +	32 (24.2)	9 (6.8)	14 (10.6)
Tumor location				
	Frontal	48 (36.4)	21 (15.9)	26 (15.2)
	Temporal	53 (40.2)	30 (22.7)	36 (27.3)
	Parietal	32 (24.2)	16 (12.1)	26 (19.7)
	Occipital	21 (15.9)	14 (10.6)	15 (11.4)
	Multifocal	57 (43.2)	29 (21.9)	36 (27.3)
	Central	98 (74.2)	42 (31.8)	54 (40.9)
	Diencefalon	11 (8.3)	4 (3.0)	7 (5.3)
Effects of steroids				
	No	8 (6.1)	3 (2.7)	4 (3.0)
	Yes	78 (59.1)	42 (31.8)	55 (41.7)
	Unknown	46 (34.8)	20 (15.2)	24 (18.2)
Midline shift				
	< 5 mm	101 (76.5)	52 (39.3)	65 (49.2)
	> 5 mm	29 (21.9)	12 (9.1)	17 (12.9)
Tumor volume				
	Median [IQR]	18.75 [9.2–41.8]		
	≤ 9.2 cm3	33 (25.0)	20 (15.9)	25 (18.9)
	9.2–18.8	38 (28.8)	18 (13.6)	25 (18.9)
	18.81–41.8	31 (23.5)	16 (12.1)	20 (15.2)
	> 41.8	33 (25.0)	10 (7.6)	15 (11.4)

Descriptive baseline characteristics of the patient cohort, including 3-month post-operative survival and completion of first-line oncological treatment

**Fig. 2:**
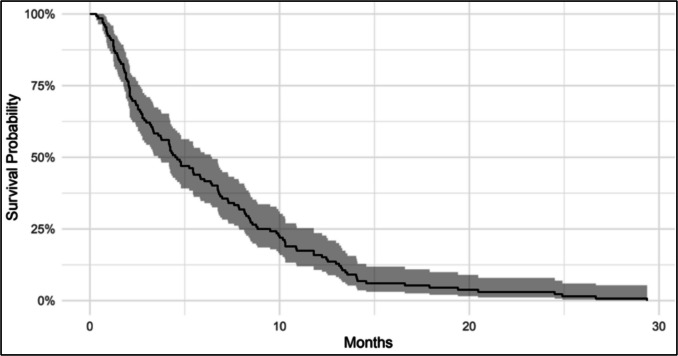
Kaplan–Meier (KM) survival curve illustrating median overall survival of 4.6 months in the entire patient cohort (*n* = 132)

The median OS was 4.6 months (95% Cl: 3.6–6.4), as shown in Fig. 2. At three months post-biopsy, 83 patients (63%) were still alive, and 22 patients (17%) remained alive at 12 months. Following biopsy, 131 patients (99%) were discussed at a MDT meeting. Oncological treatment and corresponding outcomes are summarized in Table 2. One patient died prior to the meeting*.* Of those assessed, 109 patients (83%) were recommended oncological treatment, while 22 patients (17%) were assigned to palliative care. A total of 65 patients (49%) completed Their oncological treatment plan. Median OS varied based on treatment completion. The 65 patients who completed treatment had The longest median OS at 8.3 months (95% CI: 6.4–9.9). In contrast, the 45 patients who initiated but did not complete treatment had a median OS of 3.5 months (95% CI: 2.7–4.8). Meanwhile, the 22 patients (17%) who received palliative care had The shortest median OS at 1.3 months (95% CI: 1.2–2.0). Among the 21 patients with WHO PS 3–4, The median OS was 1.6 months (95% CI: 1.2–2.5). Of These, seven patients initiated treatment and had a median OS of 1.8 months, while the 12 patients (57%) who received palliative care had a median OS of 1.3 months. Only one patient completed the full course of oncological treatment. The treatment completion status of one patient was unknown due to limited data availability.

**Table 2:** Overview of treatment completion and 3-month survival outcomes stratified by treatment type

Treatment Type	n (%)	Completed	Survival at 3 months
TMZ + RT	34 (100)	11 (32.3)	25 (73.5)
Chemotherapy (TMZ)	17 (100)	7 (41.1)	13 (76.5)
Radiotherapy (RT)	58 (100)	46 (79.3)	36 (62.1)
Palliative care	22 (100)	0 (0)	0 (0)

Radiotherapy alone had the highest completion rate (79.3%) but lower 3-month survival (62.1%). TMZ ± RT showed lower completion (32.3–41.1%) but higher survival (73.5–76.5%). No palliative care patients completed treatment nor survived beyond three months

### Preoperative factors influencing survival and treatment outcomes

After univariable analyses, WHO PS 3–4, higher NANO-scores, increasing tumor volume, and central tumor location were included in The multivariable analysis of three-month survival. WHO PS 3–4 (OR = 0.2, 95% Cl: 0.04–0.57), larger tumor volume (OR = 0.9, 95% Cl: 0.97–0.99), and central tumor location (OR = 0.3, 95% Cl: 0.09–0.87) were identified as independent negative predictors of three-month survival (Table 3).

**Table 3:** Analysis for three-months survival

	Univariable		Multivariable	
	OR (95% Cl)	*p* value	OR (95% Cl)	*p* value
Independent variabels				
Age				
≤ 75	reference			
> 76	1.541 (0.756–3.193)	0.238	1.426 (0.601–3.463)	0.424
Gender				
Female	reference			
Male	0.857 (0.416–1.749)	0.673	1.089 (0.455–2.603)	0.846
WHO				
0–2	reference			
3–4	0.095 (0.029–0.280)	< 0.001*	0.165 (0.039–0.566)	0.007*
NANO-score				
	0.793 (0.681–0.909)	< 0.01*	0.880 (0.737–1.039)	0.143
Tumor volume				
	0.978 (0.964–0.991)	< 0.01*	0.982 (0.965–0.998)	0.026*
Central				
Cortex	reference			
Central	0.211 (0.068–0.551)	< 0.01*	0.298 (0.086–0.874)	0.037*
Midline-shift				
< 5 mm	reference			
> 5 mm	0.785 (0.339–1.855)	0.573		
Effects of steroids				
No	reference			
Yes	2.391 (0.525–10.916)	0.245		
Unknown	1.090 (0.232–5.126)	0.910		
CCI	0.943 (0.779–1.145)	0.549		

Analysis of the patient cohort for survival at three-months post-operatively

^*^Significant *p*-value

In determining predictors of completing oncological treatment, the same variables – WHO PS 3–4, higher NANO-scores, increasing tumor volume, and central tumor location – were included in multivariable analysis. Among These, only WHO PS 3–4 was independently associated with a reduced likelihood of completing oncological treatment (OR = 0.06, 95% Cl: 0.003–0.32) (Table 4).

**Table 4:** Analysis for treatment incompleation

	Univariable		Multivariable	
	OR (95% Cl)	*p* value	OR (95% Cl)	*p* value
Independent variabels				
Age				
≤ 75	reference			
> 76	1.172 (0.586–2.353)	0.653	1.247 (0.542–2.894)	0.604
Gender				
Female	reference			
Male	0.544 (0.266–1.096)	0.091	0.499 (0.209–1.158)	0.109
WHO				
0–2	reference			
3–4	0.037 (0.002–0.188)	< 0.01*	0.057 (0.003–0.319)	0.008*
NANO-score				
	0.794 (0.679–0.913)	< 0.01*	0.865 (0.724–1.019)	0.093
Tumor volume				
	0.981 (0.967–0.995)	< 0.01*	0.989 (0.972–1.005)	0.177
Central				
Cortex	reference			
Central	0.338 (0.140–0.769)	< 0.05*	0.500 (0.183–1.295)	0.162
Midline-shift				
< 5 mm	reference			
> 5 mm	0.638 (0.271–1.465)	0.293		
Effects of steroids				
No	reference			
Yes	2.000 (0.458–10.306)	0.365		
Unknown	1.389 (0.302–7.449)	0.678		
CCI				
	0.941 (0.777–1.135)	0.528		

Analysis of patients unable to complete first-line oncological treatment

^*^Significant *p*-value

## Discussion

Patients with suspected GBM may be deemed unsuitable for tumor resection due to serious comorbidities or specific tumor characteristics, such as involvement of eloquent brain areas, central tumor location, or multifocal growth. Additionally, some patients are unable to initiate oncological treatment due to rapid disease progression and poor clinical status. Identifying which patients are likely to benefit from post-operative oncological therapy is important when evaluating the role of biopsy, weighing its risks against potential benefits. This study aimed to investigate survival outcomes in elderly, biopsy-only GBM-patients by identifying pre-operative prognostic factors associated with shortened survival and inability to complete oncological treatment.

Most previous studies have focused on comparing biopsy and resection in relation to OS, primarily in younger GBM patients [1, 29, 45], who typically exhibit a more favorable prognosis [13, 14, 38]. To date, research on biopsy-only gliomas in elderly populations remains limited [32]. Given the clinical challenges in this group, there is a need to identify prognostic factors to guide evidence-based treatment and personalized patient counseling. Our main findings indicate that poor WHO PS, centrally located tumors, and large tumor volumes are associated with reduced three-month survival. These findings are in agreement with previous studies reporting similar prognostic factors for poor outcomes in GBM patients [4, 21, 23].

The only independent predictor for not completing oncological treatment was preoperative WHO PS 3–4; in this subgroup, only one of 21 patients completed treatment. Patients with WHO PS 3–4 had a median OS of 1.6 months, consistent with prior studies of 2.3 months in biopsy-only GBM patients with poor PS [37]. Notably, between 18–37% of all GBM cases present with poor preoperative WHO PS [11], emphasizing its role as an important prognostic factor [23, 43]. Several studies have also proposed that PS may be a stronger predictor of survival than age, further underscoring its clinical significance [17, 23]. However, reliable data on optimal treatment strategies for patients with poor PS remain limited [7, 9, 36]. In The present study, among patients with WHO PS 3–4, the 12 patients (57%) who received palliative care had a median OS of 1.3 months, while The seven patients who initiated oncological treatment had a similar median OS of 1.8 months. This suggests that current oncological treatment may offer limited survival benefit for elderly patients with severely impaired performance status.

According to The 2021 European Association of Neuro-Oncology guidelines, obtaining a definitive histopathological diagnosis remains valuable for patient counseling, even when no further tumor-specific therapy is planned. However, the indication for biopsy may be questioned in cases where the procedure poses a high risk of complications or when the prognosis is expected to be very poor [46]. The latter is supported by a recent population-based study of 131 patients which reported that in select cases with radiologically presumed GBM and poor clinical status, biopsy could be omitted without significantly compromising patient outcomes [48].

Given the aggressive nature of GBM, there is a need to establish a definitive diagnosis rapidly, without the delays associated with histopathological analysis. Emerging non-invasive diagnostic techniques, such as radiomics and liquid biopsy, may offer safer and more tolerable alternatives in the future [20]. Prior studies report up to 93% concordance between preoperative MRI and histological diagnosis of GBM, indicating the continued diagnostic role of biopsy in unclear cases [3, 25].

A strength of our study is that the health care system in Sweden is government funded, and that there is no private practice performing brain surgery. This means that all patients residing in southern Sweden with GBM will be surgically treated at our department, reducing the risk of inclusion bias. However, there are limitations consistent with the retrospective study design. Additionally, the single-center design, cohort size, and effects of the health care system in which the study was conducted may affect the generalizability of our findings. Another limitation is the lack of detailed data on RT regimens, including duration, which limits a full assessment of treatment characteristics. Nonetheless, this does not affect our findings on treatment completion, as our primary focus was to identify preoperative factors influencing patients’ ability to adhere to regimens selected by the MDT in accordance with well-defined, standardized national guidelines [26]. Despite These limitations, our findings provide valuable insights into outcomes for GBM patients over 65 years of age who undergo biopsy only, which are an underrepresented group in clinical trials [44]. A recent study found that trial-eligible patients often exhibit better survival outcomes [19], as those with poorest prognosis are commonly excluded. This selection bias may result in overestimation of treatment benefit when trial data are generalized to elderly patients with poor PS [32]. Given this context, it is important to recognize that biopsy remains indispensable when diagnostic uncertainty exists. However, in a subgroup of patients with suspected GBM it offers no therapeutic benefit. In these cases, the risks of surgical complications, hospitalization, and psychological distress outweigh any diagnostic gain. Preoperative recognition of these patients is crucial to avoid non-beneficial invasive procedures, ensuring truly patient-centered decision-making and counseling.

## Conclusion

In this cohort of patients over 65 years with biopsy-only GBM, poor preoperative functional status, central tumor location, and larger tumor volume were significantly associated with reduced short-term survival. Furthermore, patients with poor functional status were less likely to complete oncological treatment. These findings may aid in preoperative clinical evaluation and patient counseling, helping to tailor treatment strategies to elderly GBM patients, particularly those with poor functional status. Further research is warranted to optimize management and improve outcomes in this vulnerable population.

## Data Availability

The dataset generated during and/or analyzed during the current study are available from the corresponding author on reasonable request.

## Abbreviations

ECOG/WHO PS: Eastern Cooperative Oncology Group/World Health Organization Performance Status
GBM: Glioblastoma
IDH: Isocitrate dehydrogenase
MGMT: O6-methylguanine-DNA methyltransferase
MTB: Multidisciplinary Tumor Board
NANO: Neurologic Assessment in Neuro-Oncology
OS: Overall survival
RT: Radiotherapy
TMZ: Temozolomide
